# Supplementary material for: Neural tuning instantiates prior expectations in the human visual system
Source: Nat Commun. 2023 Sep 1;14:5320. doi: 10.1038/s41467-023-41027-w (PMC10474129; doi:10.1038/s41467-023-41027-w)
Supplement: Supplementary file 1 — Supplementary Information [file 41467_2023_41027_MOESM1_ESM.pdf]

Nature Communications

Supplementary Information

Neural tuning instantiates prior expectations in the human visual system

William J Harrison, Paul M Bays and Reuben Rideaux

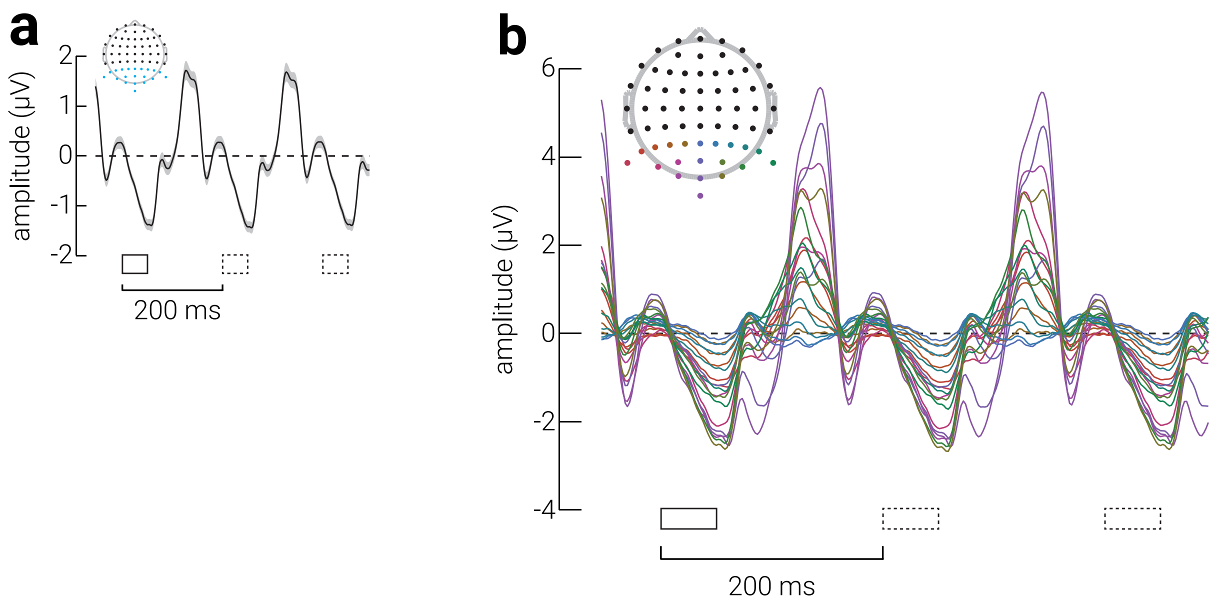

Figure S1. Event-related potentials. a) Grand average event-related potential, averaged across occipital and parietal sensors (cyan dots of inset), trials, and participants for the stimulus epoch (-50 to 500 ms). Event-locked and subsequent gratings indicated by solid and dashed black rectangles, respectively. **b**) Same as (**a**), but separately for each sensor (coloured dots of inset indicate sensor locations). Shaded regions in (**a**) indicate SEM across participants.

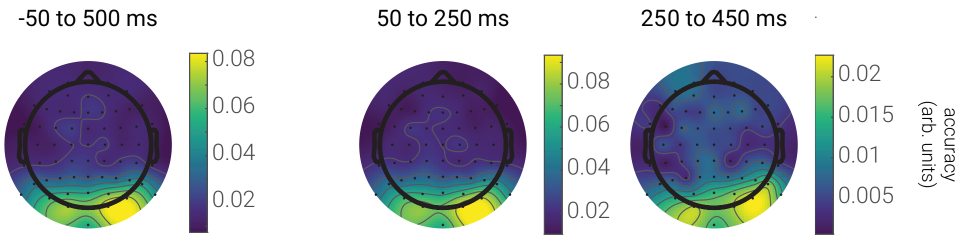

Figure S2. Topographic multivariate responses to orientations. Topographic accuracy, averaged across orientation for either the entire epoch (-50 to 500 ms), early in the epoch (50 to 250 ms) or late in the epoch (250 to 450 ms).

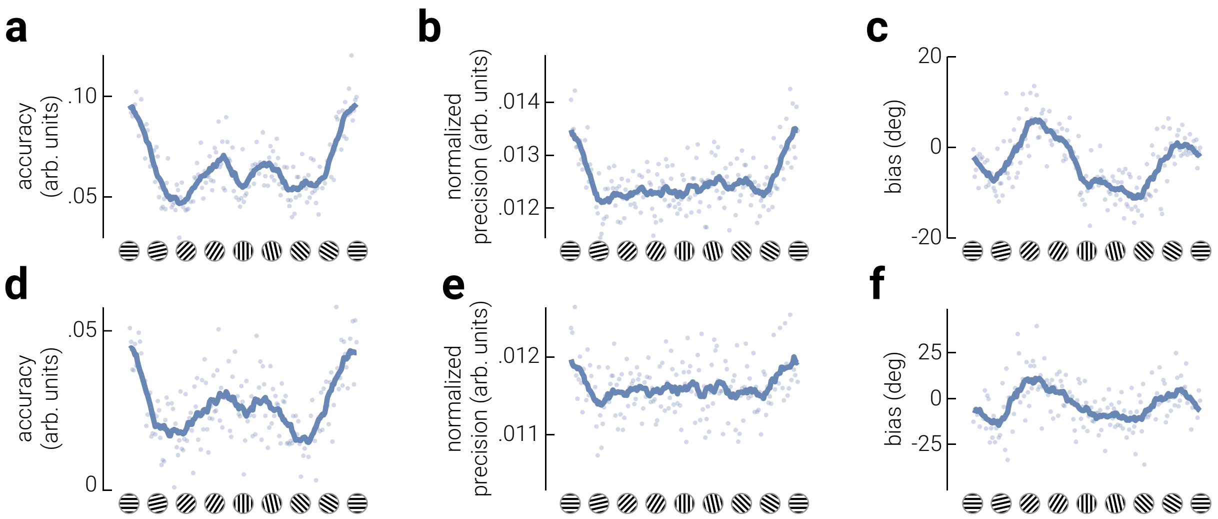

Figure S3. Temporal stability of anisotropies in the neural representation of orientation. a-c) The time-averaged (**a**) accuracy, (**b**) precision, and (**c**) bias of the inverted model responses, at all orientations, calculated from neural responses between 50 – 250 ms following stimulus onset. Cyan lines in indicate moving averages of data points (semi-transparent cyan dots). **d-f**) Same as (**a-c**), but calculated from neural responses between 250 – 450 ms following stimulus onset.

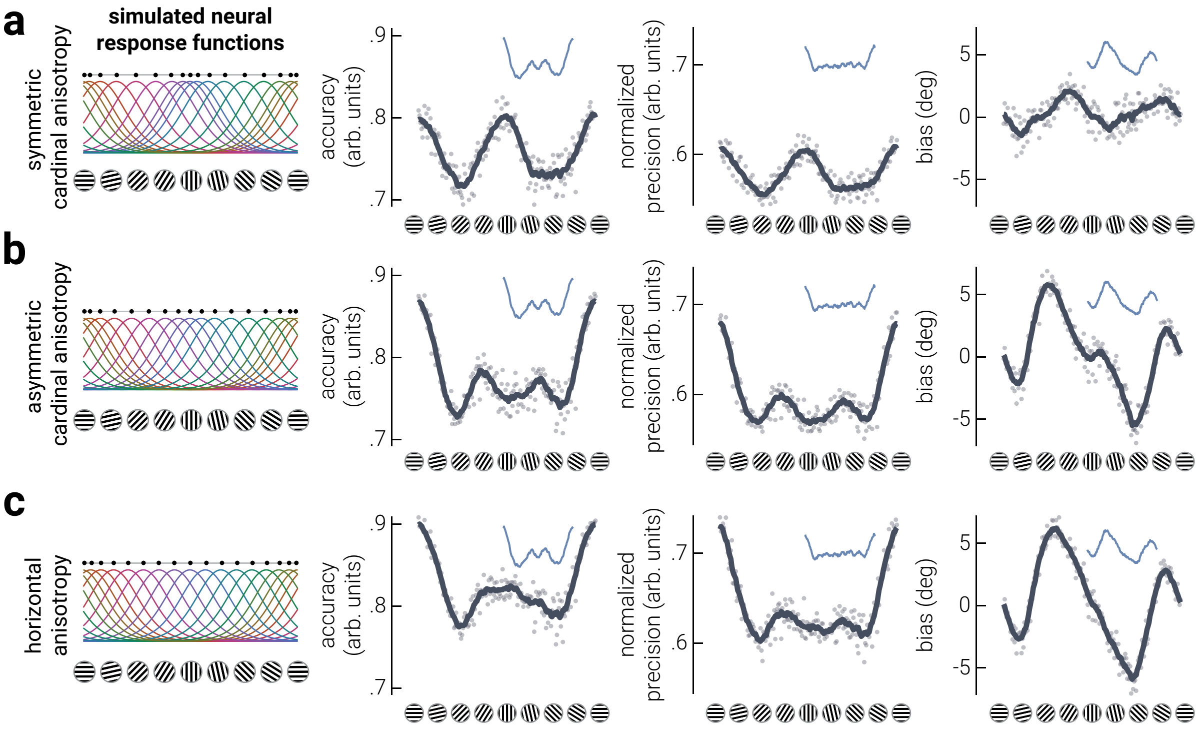

Figure S4. The influence of asymmetric cardinal tuning preferences when neural responses have high signal-to-noise. In our main simulations, we titrated the noise in the data to achieve similar signal-to-noise in the parameter estimates as in the empirical data. To reveal phenomena that may have been obscured by noise, here we reran the simulations with less noise. Results of simulations using neural response functions with (**a**) a symmetric cardinal anisotropy, (**b**) an asymmetric cardinal anisotropy, or (**c**) a horizontal anisotropy. The columns to the right are the parameter estimate results. Dark grey lines in indicate moving averages of data points (semi-transparent dots). Blue insets show the corresponding moving averages from the empirical data; horizontally distributed black dots indicate tuning preferences.
