# Supplementary material for: Neural tuning instantiates prior expectations in the human visual system
Source: Nat Commun. 2023 Sep 1;14:5320. doi: 10.1038/s41467-023-41027-w (PMC10474129; doi:10.1038/s41467-023-41027-w)
Supplement: Supplementary file 3 — Reporting Summary [file 41467_2023_41027_MOESM3_ESM.pdf]

nature portfolio

Corresponding author(s): Reuben Rideaux

Last updated by author(s): Jul 26, 2023

Reporting Summary

Nature Portfolio wishes to improve the reproducibility of the work that we publish. This form provides structure for consistency and transparency in reporting. For further information on Nature Portfolio policies, see our Editorial Policies and the Editorial Policy Checklist.

Statistics

For all statistical analyses, confirm that the following items are present in the figure legend, table legend, main text, or Methods section.

n/a	Confirmed	
☐	☒	The exact sample size (*n*) for each experimental group/condition, given as a discrete number and unit of measurement
☐	☒	A statement on whether measurements were taken from distinct samples or whether the same sample was measured repeatedly
☐	☒	The statistical test(s) used AND whether they are one- or two-sided<br>*Only common tests should be described solely by name; describe more complex techniques in the Methods section.*
☒	☐	A description of all covariates tested
☐	☒	A description of any assumptions or corrections, such as tests of normality and adjustment for multiple comparisons
☐	☒	A full description of the statistical parameters including central tendency (e.g. means) or other basic estimates (e.g. regression coefficient) AND variation (e.g. standard deviation) or associated estimates of uncertainty (e.g. confidence intervals)
☐	☒	For null hypothesis testing, the test statistic (e.g. *F*, *t*, *r*) with confidence intervals, effect sizes, degrees of freedom and *P* value noted<br>*Give P values as exact values whenever suitable.*
☒	☐	For Bayesian analysis, information on the choice of priors and Markov chain Monte Carlo settings
☒	☐	For hierarchical and complex designs, identification of the appropriate level for tests and full reporting of outcomes
☐	☒	Estimates of effect sizes (e.g. Cohen's *d*, Pearson's *r*), indicating how they were calculated

Our web collection on statistics for biologists contains articles on many of the points above.

Software and code

Policy information about availability of computer code

Data collection	EEG signals were recorded using BioSemi ActiveView v7.07
Data analysis	MATLAB v2020a; EEGLAB v2021.1; CircStat toolbox v1.21.0.0; PsychToolbox v3.0.18.13; custom code available at https://github.com/ReubenRideaux/Neural-tuning-instantiates-prior-expectations-in-the-human-visual-system

For manuscripts utilizing custom algorithms or software that are central to the research but not yet described in published literature, software must be made available to editors and reviewers. We strongly encourage code deposition in a community repository (e.g. GitHub). See the Nature Portfolio guidelines for submitting code & software for further information.

Data

Policy information about availability of data

All manuscripts must include a data availability statement. This statement should provide the following information, where applicable:

- Accession codes, unique identifiers, or web links for publicly available datasets
- A description of any restrictions on data availability
- For clinical datasets or third party data, please ensure that the statement adheres to our policy

EEG recordings have been deposited in OSF (https://osf.io/5ba9y/).

Human research participants

Policy information about studies involving human research participants and Sex and Gender in Research.

Reporting on sex and gender	Thirty-seven human adults (23 females, 14 males) participated in the experiment.
Population characteristics	Thirty-seven human adults (mean ± SD age, 23.8 ± 4.6 y) participated in the experiment.
Recruitment	Participants were recruited from The University of Queensland, had normal or corrected-to-normal vision (assessed using a standard Snellen eye chart), and were required to pass an initial screening session to qualify for the experiment (see Stimuli, Task, and Procedure section for details). Participants opted into the (paid) study; we can foresee no self-selection biases that could have meaningfully influenced the results of the current study.
Ethics oversight	The experiment was approved by The University of Queensland Human Research Ethics Committee.

Note that full information on the approval of the study protocol must also be provided in the manuscript.

Field-specific reporting

Please select the one below that is the best fit for your research. If you are not sure, read the appropriate sections before making your selection.

☒ Life sciences ☐ Behavioural & social sciences ☐ Ecological, evolutionary & environmental sciences

For a reference copy of the document with all sections, see nature.com/documents/nr-reporting-summary-flat.pdf

Life sciences study design

All studies must disclose on these points even when the disclosure is negative.

Sample size	The sample size was chosen on the basis of prior studies that showed reliable decoding with similar sample sizes, e.g., King & Wyart (2021).
Data exclusions	Data from one participant were omitted from analyses due to hardware (EEG) failure.
Replication	The results were replicated once using data from a previously published dataset, provided by different research group.
Randomization	Experimental parameters, such as the order of oriented stimuli, were randomized within and across participants.
Blinding	Investigators were not blinded during experiments because a within-subject design was used.

Reporting for specific materials, systems and methods

We require information from authors about some types of materials, experimental systems and methods used in many studies. Here, indicate whether each material, system or method listed is relevant to your study. If you are not sure if a list item applies to your research, read the appropriate section before selecting a response.

Materials & experimental systems

n/a	Involved in the study	
☒	☐	Antibodies
☒	☐	Eukaryotic cell lines
☒	☐	Palaeontology and archaeology
☒	☐	Animals and other organisms
☒	☐	Clinical data
☒	☐	Dual use research of concern

Methods

n/a	Involved in the study	
☒	☐	ChIP-seq
☒	☐	Flow cytometry
☒	☐	MRI-based neuroimaging
